# Identification of PTPN20 as an innate immunity-related gene in gastric cancer with Helicobacter pylori infection

**DOI:** 10.3389/fimmu.2023.1212692

**Published:** 2023-06-09

**Authors:** Lianjun Ma, Yang Liu, Yizhao Wang, Jiaxing Yang, Jordan Lu, Huijin Feng, Shujun Ye, Yanqing Liu

**Affiliations:** ^1^ Endoscopy Center, China-Japan Union Hospital of Jilin University, Changchun, Jilin, China; ^2^ Herbert Irving Comprehensive Cancer Center, Columbia University, New York, NY, United States

**Keywords:** gastric cancer, helicobacter pylori, prognostic value, innate immunity, tumor mutation burden

## Abstract

**Background:**

Gastric cancer (GC) is among the deadliest diseases with countless incidences and deaths each year. *Helicobacter pylori* (Hp) is the primary type of microbe that colonizes the stomach. In recent years, increasing evidence has demonstrated that Hp infection is one of the main risk factors for GC. Elucidating the molecular mechanism of how Hp leads to GC will not only benefit the treatment of GC, but also boost the development of therapeutics for other gastric disorders caused by Hp infection. In this study, we aimed to identify innate immunity-related genes in GC and investigate their potentials as prognostic markers and therapeutic targets for Hp-related GC.

**Methods:**

Firstly, we analyzed the differentially expressed innate immunity-related genes in GC samples from the TCGA database. Then prognostic correlation analysis was carried out to explore the prognostic value of these candidate genes. By combing transcriptome data, somatic mutation data, and clinical data, co-expression analysis, functional enrichment analysis, tumor mutational burden analysis, and immune infiltration analysis were performed to reveal the pathological relevance of the candidate gene. Finally, ceRNA network was constructed to identify the genes and pathways for the regulation of the candidate gene.

**Results:**

We revealed that protein tyrosine phosphatase non-receptor type 20 (PTPN20) is a significant prognostic marker in Hp-related GC. Thus, PTPN20 levels have the potential to efficiently predict the survival of Hp-related GC patients. In addition, PTPN20 is associated with immune cell infiltration and tumor mutation burden in these GC patients. Moreover, we have also identified PTPN20-related genes, PTPN20 protein-protein interactions, and the PTPN20 ceRNA network.

**Conclusion:**

Our data suggest that PTPN20 may have critical functions in Hp-related GC. Targeting PTPN20 may be a promising way to treat Hp-related GC.

## Introduction

Gastric cancer (GC) is a malignant cancer type that occurs in the stomach. According to a recent study, GC is a global threat and has the highest incidence rate in Eastern Asia (32.5 incidences in men and 13.2 in women per 100,000 persons) (1). Experts estimate that there may have been over one million new GC cases and 769,000 GC related deaths in 2020. More than 90% of GC cases are adenocarcinomas, composed of two major subtypes: the intestinal type and the diffuse type (2). Although the development of therapies has improved the overall survival rate of GC patients, the prognosis for advanced GC is still low. Thus, it is necessary to identify the risk factors of GC and to elucidate the pathological process of GC transformation and progression (3, 4). Unhealthy dietary habits are the major risk factor of GC, including high salt diets, low intake of fruits and vegetables, alcohol consumption, and smoking. Apart from dietary factors, *Helicobacter pylori* (Hp) infection has been progressively recognized to be a cause for GC.

While Hp is the primary type of bacteria that colonizes the stomach, its existence and pathological relevance has long been neglected by scientists and clinicians. The seminal discovery of Warren and Marshall in the early 1980s (5, 6) established Hp as one of the most prominent and well-studied bacterial species of stomach diseases. Hp infection can have several effects on stomach mucosa that manifest in different pathological disorders, including gastritis, gastric/duodenal ulcers, intestinal metaplasia, and eventually GC (7–9). Poor socio-economic conditions, particularly during childhood, are the main risk factors of Hp infection. Food safety, clean water, and the adoption of individual servings can effectively reduce the chances of infecting or spreading Hp. Due to growing scientific and medical education, there is increasing attention towards Hp prevention, testing and treatment. However, Hp infection currently still poses a global threat. Given the fact that Hp is the central cause for diverse stomach disorders (including GC), developing effective therapeutics to ameliorate its symptoms and to eradicate it entirely is paramount. Such therapeutics hinge on the understanding of the molecular and cellular changes in the gastric environment where Hp infection occurs. Hp is long believed to be an extracellular bacterium. However, there is evidence demonstrating that Hp can be invasive and may be a facultative intracellular organism (10). In either case, Hp can trigger innate immune responses (11, 12).

Innate immunity is the first line of defense protecting the host from pathogen infection. Various immune cell types, such as phagocytes (macrophages and neutrophils), dendritic cells, and natural killer (NK) cells, work together to build this system (13). In particular, innate immunity plays a vital role in defending the body against bacterial infections, like Hp (11, 12). In each stage of Hp-induced stomach disorders, there are complex interactions between Hp and the innate immune system. On the one hand, different types of innate immune cells are orchestrated to inhibit Hp proliferation and eliminate it. These cells also engage in crosstalk with the adaptive system to exert a synergistic effect on controlling Hp. On the other hand, Hp has evolved versatile ways to evade the attack from innate immune cells. At the molecular level, Hp infection changes the gene expression profile of both innate immune cells and cells of the stomach. The altered gene expression pattern in Hp-related GC cells can reveal how these cells are influenced by Hp and which gene/pathway we can target to treat this type of GC. In addition, the differentially expressed genes may also have prognostic value.

In this current study, we analyzed the innate immunity-associated and differentially expressed genes in normal and GC (with or without Hp infection) cells and identified that protein tyrosine phosphatase non-receptor type 20 (PTPN20) is a good candidate for prognosing Hp-related GC patients. We also identified a PTPN20-associated gene signature and analyzed several basic PTPN20-related characteristics (protein-protein interactions, the immune cell infiltration, tumor mutation burden, and the ceRNA network). Interestingly, PTPN20 was found to be tightly related to the function of many innate immune cells.

## Methods

### Data acquisition

Transcriptome profiles, somatic mutation information and the corresponding clinical data of 448 gastric cancer (GC) cases (normal samples, 36 cases; tumor samples, 306 cases, of which 20 cases of GC with Hp infection, 157 cases without Hp infection) were retrieved from the TCGA database (https://tcga-data.nci.nih.gov/tcga) from dbGaP with the research accession number phs000178.v11.p8. GSE60427 was obtained from the GEO database (accession number: GSE60427). Innate Immune System SuperPath-related gene sets (2024 Genes in total) were downloaded from the PathCards database (https://pathcards.genecards.org/), of which 3 Pathways in the Innate Immune System SuperPath are Immune System, Neutrophil degranulation, and Innate Immune System.

### Differential expression analysis

The limma package (14) (https://bioconductor.org/packages/limma/) was utilized to analyze the mRNA expression matrix between GC with Hp infection samples and normal samples, as well as GC with Hp infection and non-infection. In the analysis of the differential expression between GC samples with Hp infection and normal samples, the criteria for differentially expressed genes (DEGs) were |Log 2 (Fold Change)| > 2 and p-value < 0.05, while for the Hp infection and non-infection groups, the criteria for DEGs were |Log 2 (Fold Change)| > 1 and p-value < 0.05. Afterwards, we took the intersection between these two DEGs.

### Prognostic correlation analysis

Survminer and survival packages (R/Bioconductor) from R software were used for survival analysis for the intersection gene sets. Samples with incomplete clinical information should be discarded. The survival curve was created using the Kaplan-Meier method (15). The statistical significance was determined using log rank, with the significant p-value threshold set at 0.05. Subsequently, Cox regression was used for univariate analysis of PTPN20 and clinical traits to assess the association between these factors and prognosis. Lastly, the survival ROC package of the R software was used to draw the ROC curve to validate the prognosis.

### Identifying genes related to PTPN20

First, the genes related to PTPN20 were obtained based on the coexpression analysis using Pearson correlation coefficients (coefficients > 0.60, P < 0.001). Then, differentially expressed genes were identified by comparing the high- and low-expression of PTPN20 by differentiation analysis using package limma. DEGs that met the following criteria were considered significant: |Log 2 (Fold Change)| > 1 and p-value < 0.05. The Protein-protein interaction (PPI) analysis of differential genes was carried out using the STRING database and the PPI network model was created using Cytoscape.

### Functional enrichment analysis of DEGs

Gene ontology (GO), Kyoto encyclopedia of genes (KEGG) (www.kegg.jp) and genomes enrichment analyses (GSEA) (https://www.broadlnstitute.org/gsea/) of DEGs were conducted using R packages cluster Profiler (16, 17). Subsequently, enrich plot was used to explore the biological functions and signaling pathways and ggplot2 was used to plot the results (http://ggplot2.org).

### Immune cell infiltration landscape analysis

Using the R package, CIBERSORT (https://cibersort.stanford.edu/) was used to estimate the relative fractions of 22 infiltrating immune cell types in each tumor sample. We then evaluated the association between the results of the immune infiltration distribution and PTPN20 expression.

### Tumor mutation burden estimation

Tumor mutation burden (TMB) is defined as the total number of exonic mutations per mega base of tumor DNA. The TCGA biolinks package in R was used to download mutation annotation files (18). Subsequently, to distinguish the genetic characteristics of GC patients with varying expressions of TPTN20, we utilized the maftools package to generate a mutation annotation format (MAF) based on data from the TCGA database.

### Construction of ceRNA network

Diana-microt (http://diana.cslab.ece.ntua.gr/microT/), ElMMo (http://www.mirz.unibas.ch/ElMMo2/), MicroCosm (www.ebi.ac.uk/enright-srv/microcosm/) and miRanda (https://tools4mirs.org/software/target_prediction/miranda/) databases were used to predict the target miRNAs of PTPN20. The miRNAs that bound to mRNAs were the ones that came from the overlapping parts of at least three data sets and also intersected with the differentially expressed miRNAs (DEmiRNAs). The DIANA-LncBase v3 (https://diana.e-ce.uth.gr/lncbasev3) online tool was used to predict the lncRNAs that bind to miRNAs.

Finally, using the DElncRNA-DEmiRNA and DEmiRNA-DEmRNA connections, we built a coexpression network of differentially expressed RNAs. Cytoscape (version 3.9.0) was used to visualize the competing endogenous RNA (ceRNA) network.

## Results

### Identification of PTPN20 as a hub gene associated with Hp-related GC

**Figure 1:**
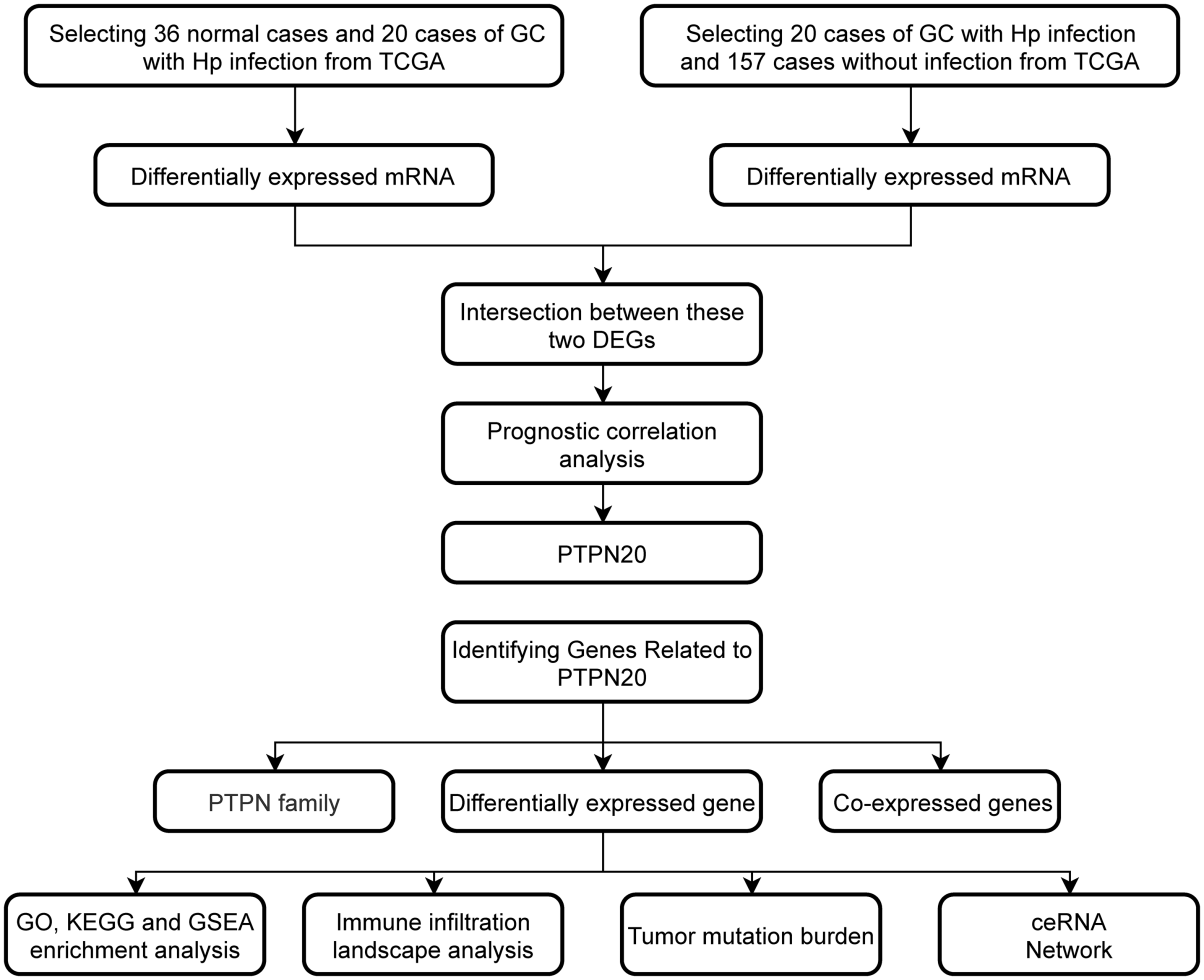
Workflow of the study design.

**Figure 2:**
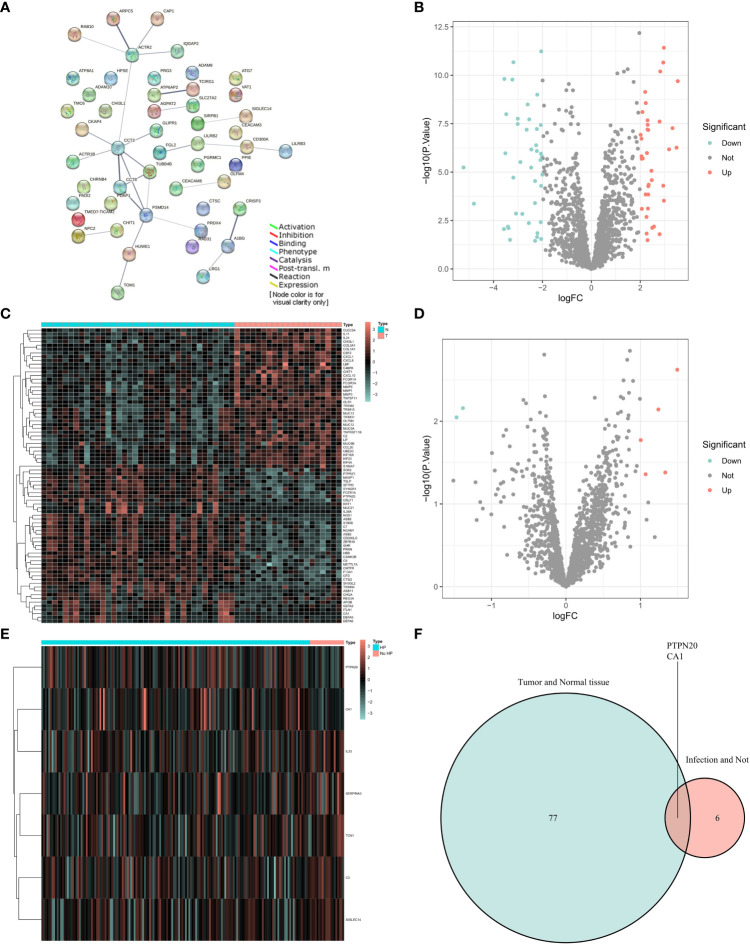
Identification of PTPN20 as a hub gene associated with Hp-related GC. **(A)** A gene network analysis of Innate Immune System SuperPath-related genes by using STRING. **(B)** A Volcano plot of differentially expressed genes between GC with Hp infection samples and normal samples. **(C)** A heatmap of differentially expressed genes between GC with Hp infection samples and normal samples. **(D)** A Volcano plot of differentially expressed genes between GC with Hp infection and non-infection. **(E)** A heatmap of differentially expressed genes between GC with Hp infection and non-infection. **(F)** Venn diagram of the intersection of the two differentially expressed gene sets.

Our experimental workflow is shown in **Figure 1**. To understand the interactions of Innate Immune System SuperPath-related genes, we performed a gene network analysis of these genes by using STRING (**Figure 2A**). As illustrated by the volcano plots, the gene expression profiles identified 78 differentially expressed genes in GC samples with Hp infection, with 37 genes upregulated and 41 genes downregulated when compared to the expression in normal control samples (**Figures 2B, C**). Between GC with Hp infection and non-infection groups, 7 differentially expressed genes were identified, including 2 down-regulated genes and 5 upregulated genes (**Figures 2D, E**). The two datasets showed an overlap of 2 genes: PTPN20 and CA1, respectively (**Figure 2F**). Compared to the normal control group and the GC group without Hp infection, the expressions of PTPN20 and CA1 are significantly reduced in the Hp-related GC group, with logFC values of -2.45 and -1.47 for PTPN20, and -2.21 and -1.38 for CA1, respectively.

### Prognostic correlation analysis of PTPN20 in Hp-related GC

**Figure 3:**
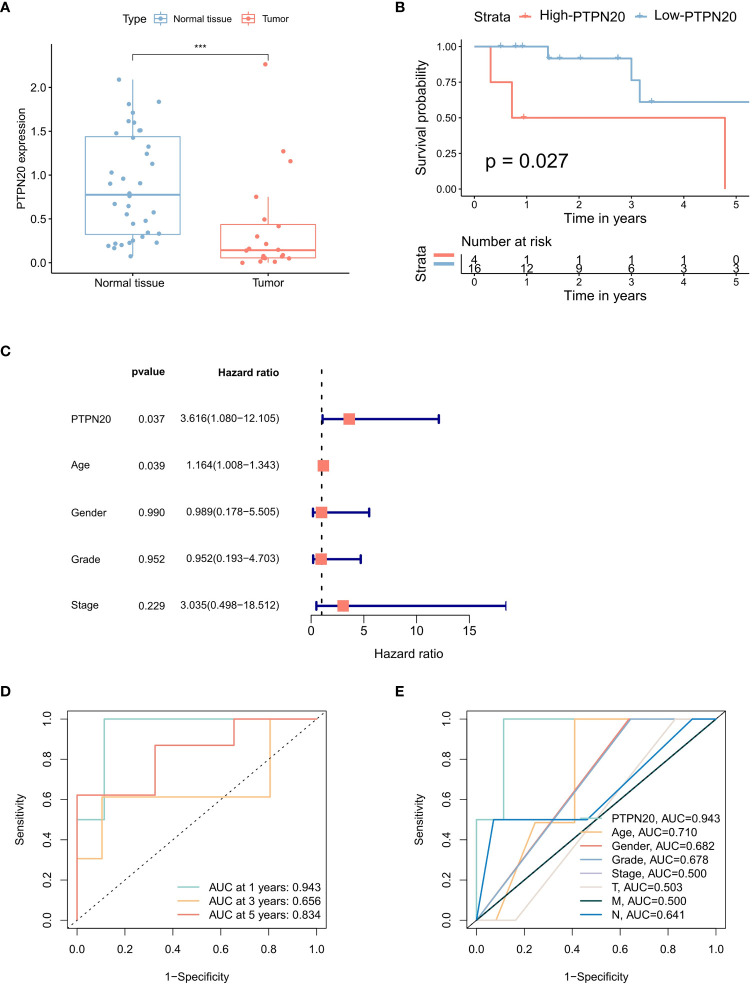
Prognostic correlation analysis of PTPN20 in Hp-related GC. **(A)** The PTPN20 expression in the Hp-related GC samples and the normal samples. **(B)** Kaplan-Meier survival distributions of PTPN20 in GC with Hp infection samples. **(C)** A forest plot for risk factors for Hp-related GC patients from univariate analysis. **(D)** Time-dependent ROC curves of overall survival at 1, 3- and 5- years. **(E)** ROC curves for clinical characteristics in patients with GC with Hp infection samples. ***, p < 0.001.

The results indicated that the PTPN20 expression in Hp-related GC samples were remarkably lower than the normal samples by the Wilcoxon rank sum test (**Figure 3A**). We also validated our result by using external patient data. In the GSE60427 dataset, tissue with Hp infection had lower levels of PTPN20 expression than non-infected tissue, as shown in **Supplemental figure 1**. Furthermore, our results suggest that elevated PTPN20 expression is a significant predictor of poor prognosis in Hp-related GC patients, as our Kaplan-Meier survival analysis revealed a significant association between high PTPN20 expression and poor prognosis (p <0.05), as presented in **Figure 3B**. Subsequently, by conducting univariate Cox analysis, we found that high PTPN20 expression was significantly associated with a higher risk of overall survival (hazard ratio [HR] = 3.62, p = 0.037) (**Figure 3C**). To validate these findings, we performed ROC curve analysis of PTPN20 gene expression data (**Figure 3D**). The areas under the ROC curve were 0.943 (1-year ROC), 0.656 (3-year ROC), and 0.834 (5-year ROC), indicating that PTPN20 expression has high diagnostic accuracy in predicting Hp-related GC prognosis. Clinical characteristic analysis showed that the diagnostic value of PTPN20 gene was significantly higher compared to various clinical features (**Figure 3E**), with an AUC value of 0.710 for age, 0.682 for gender, 0.678 for the grade, and 0.500 for Stage. To summarize, our findings support the hypothesis that PTPN20 plays a critical role in cancer progression and may serve as a prognostic biomarker for Hp-related GC patients.

### Identification of genes related to PTPN20

**Figure 4:**
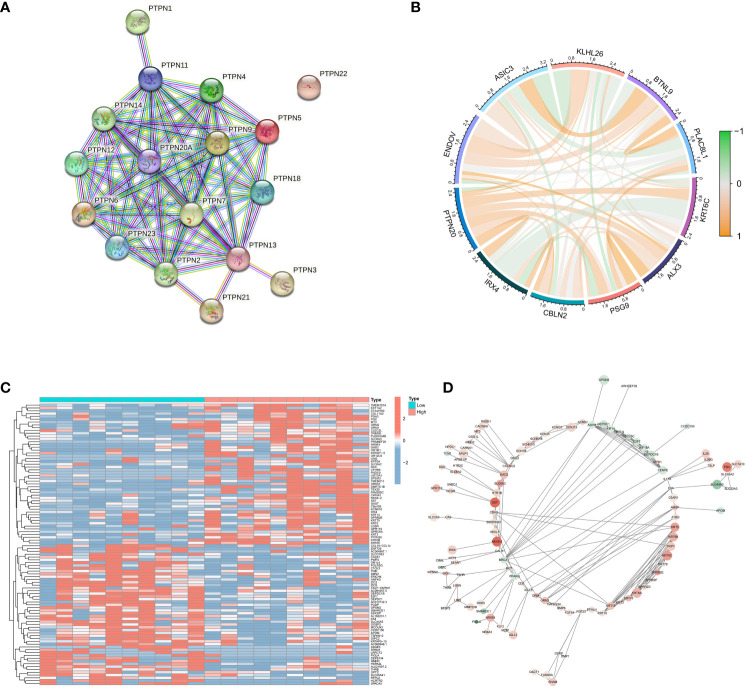
Identification of genes related to PTPN20. **(A)** The PPI network of the PTPNs family genes. **(B)** Correlation between PTPN20 expression and 10 selected genes. **(C)** Heatmap showing differentially expressed genes with high and low expression of PTPN20. **(D)** The PPI network of these differentially expressed genes.

PTPN20 is a member of the PTPNs gene family. STRING database (https://string-db.org/) was used to construct the PPI network to understand the connections among the PTPNs family genes (**Figure 4A**). It indicated the tight correlation and regulation relationship of these genes. In addition, the PTPNs gene family, which is a family of protein tyrosine phosphatases, has many members: PTPN1, PTPN2, PTPN3, PTPN4, PTPN5, PTPN6, PTPN11, PTPN20, and so on. PTPNs have received a great deal of attention due to their roles in cell growth, apoptosis, and signal transduction pathways. It has also been discovered that PTPNs play an important role in the development of various cancers, including digestive tract tumors (19–23). PTPN11, for example, has been linked to pancreatic cancer (24), and PTPN4 has been linked to breast cancer (25). PTPN6 has also been linked to bladder cancer progression (26). The coexpression analysis of PTPN20 is then performed using Pearson correlation analysis. The P-value and correlation coefficient value was used to select 5 genes that are each positively and negatively correlated with PTPN20 expression, and visualization was done in R studio using the corrplot package (**Figure 4B**). Notably, most of them were related to the development of digestive system tumors. Specifically, a study discovered that patients with colon cancer had a worse prognosis when their blood levels of BTNL9 were greater (27), and IRX4 may play important roles in the development and progression of gastric cancer. The limma package was used to analyze the differentially expressed genes between groups with high and low expression of PTPN20, with the screening criteria of P < 0.05 and |logFC| ≥ 1. A total of 294 differentially expressed genes (223 upregulated, 71 downregulated) were screened (**Figure 4C**), and PPI analysis was performed using STRING (version 10.0, http://string-db.org) (**Figure 4D**).

### Functional enrichment analysis of DEGs

**Figure 5:**
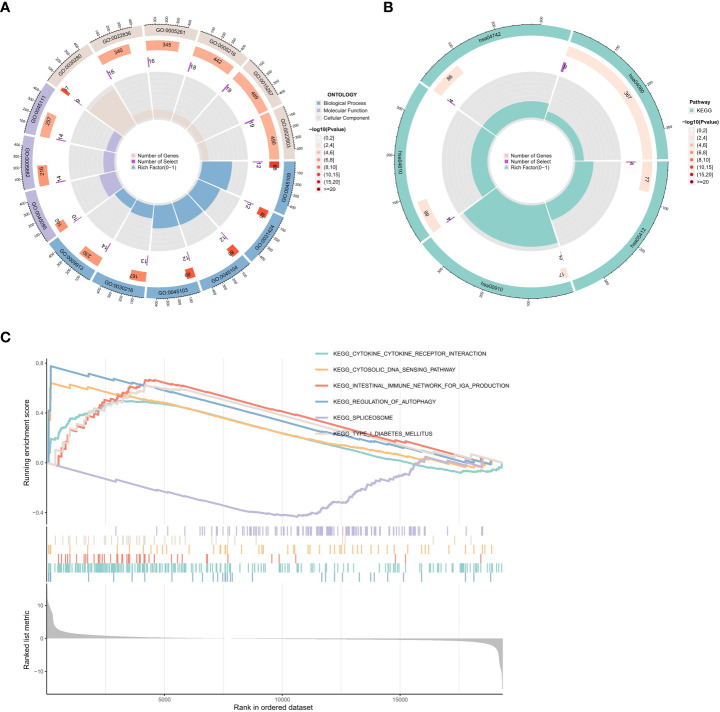
Functional enrichment analysis of DEGs. **(A)** Results of GO enrichment analyses. **(B)** Results of KEGG enrichment analyses. **(C)** Results of GSEA enrichment analyses.

To investigate the potential biological functions of the common DEGs, we used the GO term (**Figure 5A**), KEGG pathway (**Figure 5B**), and GSEA analyses (**Figure 5C**).

Biological process (BP), molecular function (MF), and cellular component (CC) are among the GO annotation results. The results revealed that the BP is primarily associated with the development of the skin, including keratinocyte differentiation, epidermal cell differentiation, epidermis development, and skin development. For MF analysis, DEGs are primarily enriched in skin epidermis structural constituents, cytoskeleton structural constituents, ligand-gated cation channel activity, ligand-gated ion channel activity, and ligand-gated channel activity. The results of the CC enrichment analysis revealed that DEGs played a significant role in the keratin filament, intermediate filament, and intermediate filament cytoskeleton (**Figure 5A**).

The KEGG pathway analysis showed that the DEGs were significantly enriched in the arrhythmogenic right ventricular cardiomyopathy, complement, and coagulation cascades, taste transduction, and neuroactive ligand-receptor interaction (**Figure 5B**).

We also used GSEA to evaluate the potential signaling pathway of PTPN20 in Hp-related GC. We found some signaling pathways that may be related to the Hp infection, including Toll-like receptor signaling pathway, cytosolic DNA sensing pathway, RIG-I-like receptor signaling pathway, and antigen processing and presentation pathway. There are also some pathways related to innate immunity, such as (1) KEGG Toll-like Receptor Signaling Pathway. (2) KEGG Cytosolic DNA Sensing Pathway. (3) KEGG RIG-I-like Receptor Signaling Pathway. (4) KEGG Antigen Processing and Presentation. (5) KEGG Inflammasome. (6) KEGG NOD-like Receptor Signaling Pathway. (7) KEGG Complement and Coagulation Cascades. (8) KEGG Natural Killer Cell Mediated Cytotoxicity. Our results illustrate the complex mechanism of PTPN20 in Hp-related GC while also clarifying the potential innate immune biological pathways that PTPN20 may contribute to in Hp-related GC (**Figure 5C**).

### Immune cell infiltration landscape analysis

**Figure 6:**
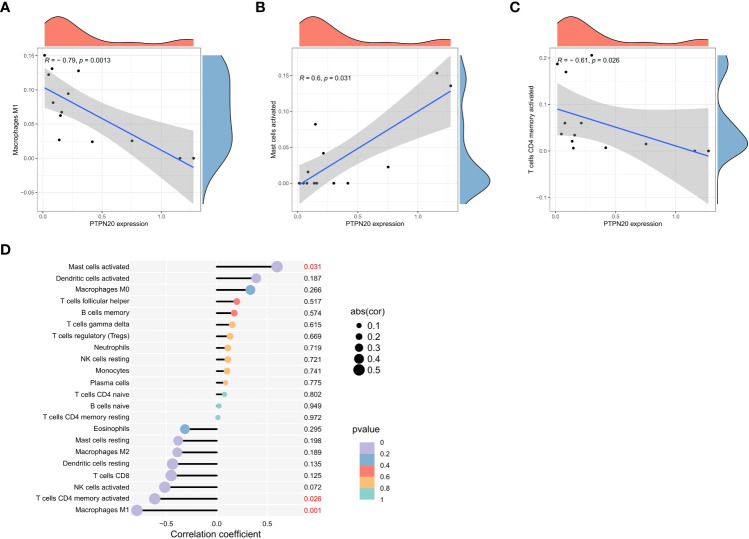
Immune cell infiltration landscape analysis. **(A–C)** PTPN20 expression was negatively correlated with Macrophages M1 and T cells CD4 memory activated, and positively correlated with Mast cells activated. **(D)** The relationship between the outcomes of immune infiltration distribution and PTPN20 expression.

PTPN20 expression was found to have a significant negative residual correlation with the levels of Macrophages M1 and activated mast cells, as well as a significant positive correlation with activated CD4 T cells (**Figures 6A–D**). Notably, Macrophages M1 and mast cells play a vital role in innate immunity. M1 macrophages have been shown to provide an antitumoral immune response in gastric cancer. They release cytokines, including IL-6, IL-12, IL-8, and TNF that stimulate type I T helper cells to kill tumors (28). These data suggested that PTPN20 has a role in regulating innate immunity in Hp-related GC.

As the tumor microenvironment (TME) plays a crucial role in tumor progression, we calculated the TME score for each GC samples. Then, we obtained TME scores for every GC sample, including ImmuneScore, StromalScore, and ESTIMATEScore, and found that the three scores in the PTPN20 low expression group were lower than those of the high expression group (**Supplemental Figure 2**). This result indicates that PTPN20 expression is associated with the immune and stromal components of the TME, which may have implications for prognosis and response to therapy in GC patients.

### Tumor mutation burden estimation

**Figure 7:**
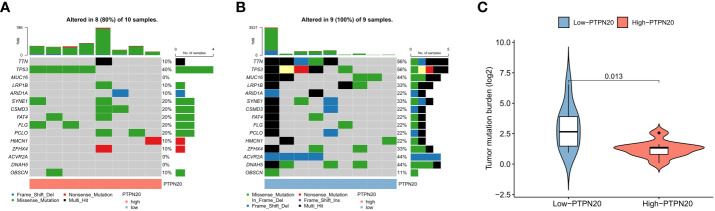
The relationship between TMB and the expression of PTPN20. **(A, B)** The oncoplots of the mutation genes in Hp-related GC patients for the high and low PTPN20 expression groups. **(C)** Higher TMB levels correlated with low expression of PTPN20.

Somatic mutation data was analyzed and visualized in the PTPN20 high and low expression groups because TMB has been linked to immunotherapeutic response and prognosis in cancer. **Figures 7A, B** show the top 15 most frequently mutated genes in these two cohorts. Notably, mutations in TTN, MUC16, ACVR2A, and DNAH5 were significantly lower in the low-expression group than in the high-expression group; this suggests that a higher PTPN20 expression level may be indicative of a lower tumor mutation burden (**Figure 7C**). Furthermore, compared to the wild-type subgroup, the TTN mutant subgroup was substantially related to a better prognosis, a higher TMB, and a better response to immune checkpoint blockade in solid tumors, according to a prior study (29, 30).

### Construction of ceRNA network

**Figure 8:**
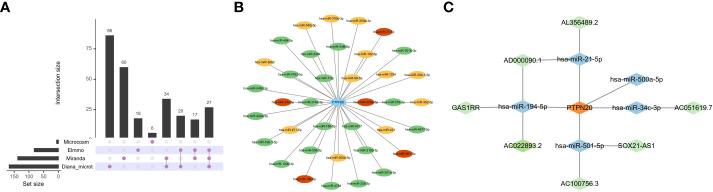
Construction of ceRNA Network. **(A, B)** The ceRNA network’s target miRNAs were predicted based on the Diana-microt, ElMMo, MicroCosm, and miRanda databases. Green indicates that the miRNA is only found in one database, yellow suggests that it is present in two databases, and red indicates that it is present in three databases. **(C)** Network of ceRNA interactions. Blue represents miRNAs, and green represents lncRNAs.

The relationship between PTPN20, down-regulated DElncRNAs, and upregulated DEmiRNAs was predicted using bioinformatics methods. The ceRNA network’s target miRNAs were predicted using the Diana-microt, ElMMo, MicroCosm, and miRanda databases (**Figures 8A, B**). These target genes were the miRNAs that were in the ceRNA network after intersecting with DEmiRNAs. They are broadly associated with gastric cancer progression, such as, by targeting SMAD7 and activating the TGF-/Smad pathway, exosomal miR-21-5p promotes mesothelial-to-mesenchymal transition (MMT) in peritoneal mesothelial cells (PMCs) and peritoneal metastasis of gastric cancer (31).

The DIANA-LncBase v3 web tool (https://diana.e-ce.uth.gr/lncbasev3) was used to predict lncRNAs that bind to miRNAs. The DElncRNAs were then intersected with the lncRNAs collected from the DIANA-LncBase to obtain the lncRNAs in the ceRNA network.

We constructed a ceRNA network with 7 lncRNAs and 5 miRNAs. Then, we used Cytoscape software to visualize the network, as shown in **Figure 8C**.

## Discussion

PTPN20 belongs to the protein tyrosine phosphatases (PTPs) super family (32, 33). This family has tens of members, which are characterized by their ability to remove phosphate groups from tyrosine residues on their substrates–an opposite reaction to protein tyrosine kinases (PTKs). Some PTPs are also able to act on phosphothreonine and phosphoserine residues. The PTP super family has been extensively studied and demonstrated to be closely linked to both normal cellular activities as well as many diseases. Thus, it is reasonable to assume that PTPN20 also participates in the regulation of normal and pathological processes. However, the precise function and mechanism of PTPN20 remains poorly understood. In 2005, a group from Australia reported that the human PTPN20 gene is located at the chromosome locus 10q11.2 and generates many isoforms by alternative splicing (34). One of these isoforms, hPTPN20a, was shown to be targeted to sites of actin polymerization, suggesting that hPTPN20a may be involved in membrane mobility regulation. In zebrafish, Overman and Hertog found that Ptpn20 is a multi-domain PTP enzyme (35). The same group knocked down Ptpn20 expression in zebrafish embryos and observed defects in convergence and extension cell movements, which could be rescued by dominant negative RhoA (36). This result is in consistent with the idea that PTPN20 participates in the regulation of cell skeleton structure and movement (34). In hydrocephalic H-Tx rat, knocking out the Ptpn20 gene increases the level of phosphorylated NKCC1, which is a Na-K-Cl cotransporter (37). This may cause overproduction of cerebrospinal fluid and lead to the development of hydrocephalus. In peripheral arterial occlusive disease, PTPN20 was found to be downregulated in intermediate lesions when compared to normal femoral arteries, indicating that restoring PTPN20 may improve peripheral arterial occlusive disease (38). In hepatocellular carcinoma (HCC), PTPN20 may act as an inhibitor for the STAT3 signaling pathway (39). A microRNA miR-589-5p promotes HCC cell stemness and chemoresistance, partly by inhibiting PTPN20 expression. By using the microarray-based comparative genomic hybridization, Yildirim et al. tried to identify the chromosome alterations in pediatric cancers (40). They revealed that PTPN20 may play a role in Ewing sarcoma (ES)/primitive neuroectodermal tumor (PNET) as its gene locus underwent deletion in some ES/PNET samples.

PTPN20 has been shown to have differential expression across cancer types, suggesting that it may have distinct roles in different cancer types. Specifically, while the expression of PTPN20 was reduced in colorectal cancer (19), PTPN20 levels showed no change between acute myeloid leukemia cell and normal cells (41). In esophageal carcinoma, PTPN20 was upregulated and associated with both the TNM stage and N stage. Importantly, high levels of PTPN20 predicted poor survival in esophageal carcinoma patients, indicating that PTPN20 levels may also have prognostic value in certain cancer types. Moreover, PTPN20 can also function as cancer marker or antigen. Condomines et al. utilized gene expression profiling and real-time PCR to identify possible cancer-testis antigens and found PTPN20 was a promising candidate in multiple myeloma and bladder cancer (42). Similarly, another group found PTPN20 to be a marker for multiple myeloma by using a self-training subspace clustering (SSC)-low-rank representation (LRR) algorithm (43). PTPN20 is also involved in the immune system processes of various diseases. In adalimumab-treated rheumatoid arthritis patients, Chen et al. performed an immune-related microarray and identified PTPN20 along with other 7 genes that could serve as a biomarker for anti-drug antibody development and EULAR response (44). Wang et al. carried out a thorough analysis of the PTP family members in breast cancer (45). They uncovered that the expression of PTPN20, PTPN7, and several other PTPs were significantly augmented in breast cancer tissues compared to normal tissues and identified a strong correlation between PTPN7 expression and immune infiltration. Interestingly, Gandhi et al. reported that PTPN20 and PTPN7 have similar structures (46), which implies that PTPN20 is also associated with immuno-hot tumors. Yet despite these many advancements in uncovering the versatile functions of PTPN20, its function and application in GC remains elusive.

As a life-threatening malignancy, GC is worthy of more attention and research efforts. While healthy dietary practices are undoubtedly conducive to decreasing GC incidence, the role of Hp infection should not be underestimated. Hp testing is an effective way to diagnose its infection. Identifying the key genes in Hp-associated GC will not only boost our understanding of the mechanism underlying GC progression but will also provide molecular targets for novel therapeutics and drugs. The development of big-data techniques makes it possible to perform bioinformatic analyses by comparing numbers of GC samples (with or without Hp infection). Innate immunity is the first and primary way that the body fights Hp. By identifying the differentially expressed, innate immunity-related genes in GC samples with Hp infection, we can illuminate more promising targets. Indeed, in this study we have found a novel gene, PTPN20, which is downregulated in Hp-related GC samples and associated with the innate immune response. PTPN20 is effective in predicting the survival of GC patients. Additionally, PTPN20 and its related gene set was found to be associated with several critical characteristics of GC, such as immune cell functions and tumor mutation burden. Moreover, the PPI and ceRNA network centered by PTPN20 may offer targets for future work on PTPN20 and Hp-associated GC.

Together, we have identified a differentially expressed, innate-immunity associated PTPN20 gene, which has prognostic value in gastric cancer. Through future investigation of PTPN20 in GC, we hope to develop new therapeutics for GC by targeting this gene.

## Data availability statement

Publicly available datasets were analyzed in this study. This data can be found here: dbGaP accession number phs000178.v11.p8.

## Author contributions

LM and YQL conceived the project. LM, YL, and YW analyzed the data. YQL, YL, and JL wrote the manuscript. JY, HF, and SY reviewed and revised the manuscript. The authors read and approved the final manuscript. The requirements for authorship have been met. Each author believes that the manuscript represents honest work. All authors contributed to the article and approved the submitted version.

## Supplementary material

Supplementary Figure 1 PTPN20 expression in the GSE60427 dataset. Tissues with Hp infection had lower levels of PTPN20 expression than non-infected ones.

Supplementary Figure 2 The relationship between PTPN20 expression and tumor microenvironment scores. The PTPN20 low expression group had lower immuneScore, StromalScore, and ESTIMATEScore compared to the high expression group.
