# Supplementary figures and images for: Identification of PTPN20 as an innate immunity-related gene in gastric cancer with Helicobacter pylori infection
Source: Front Immunol. 2023 Jun 9;14:1212692. doi: 10.3389/fimmu.2023.1212692 (PMC10287967; doi:10.3389/fimmu.2023.1212692)

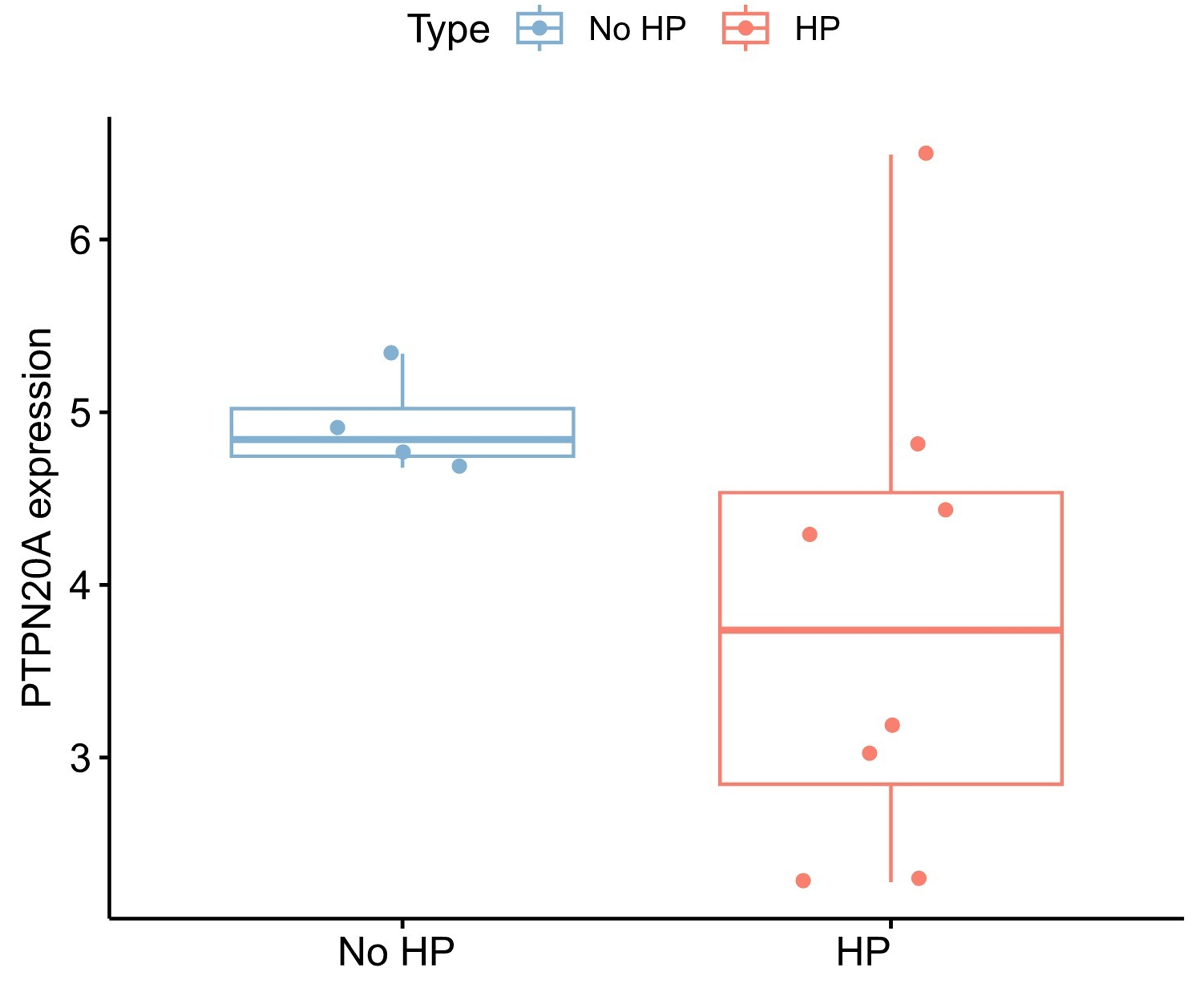

Supplement: Supplementary Figure 1 — PTPN20 expression in the GSE60427 dataset. Tissues with Hp infection had lower levels of PTPN20 expression than non-infected ones. [file Image_1.tif]

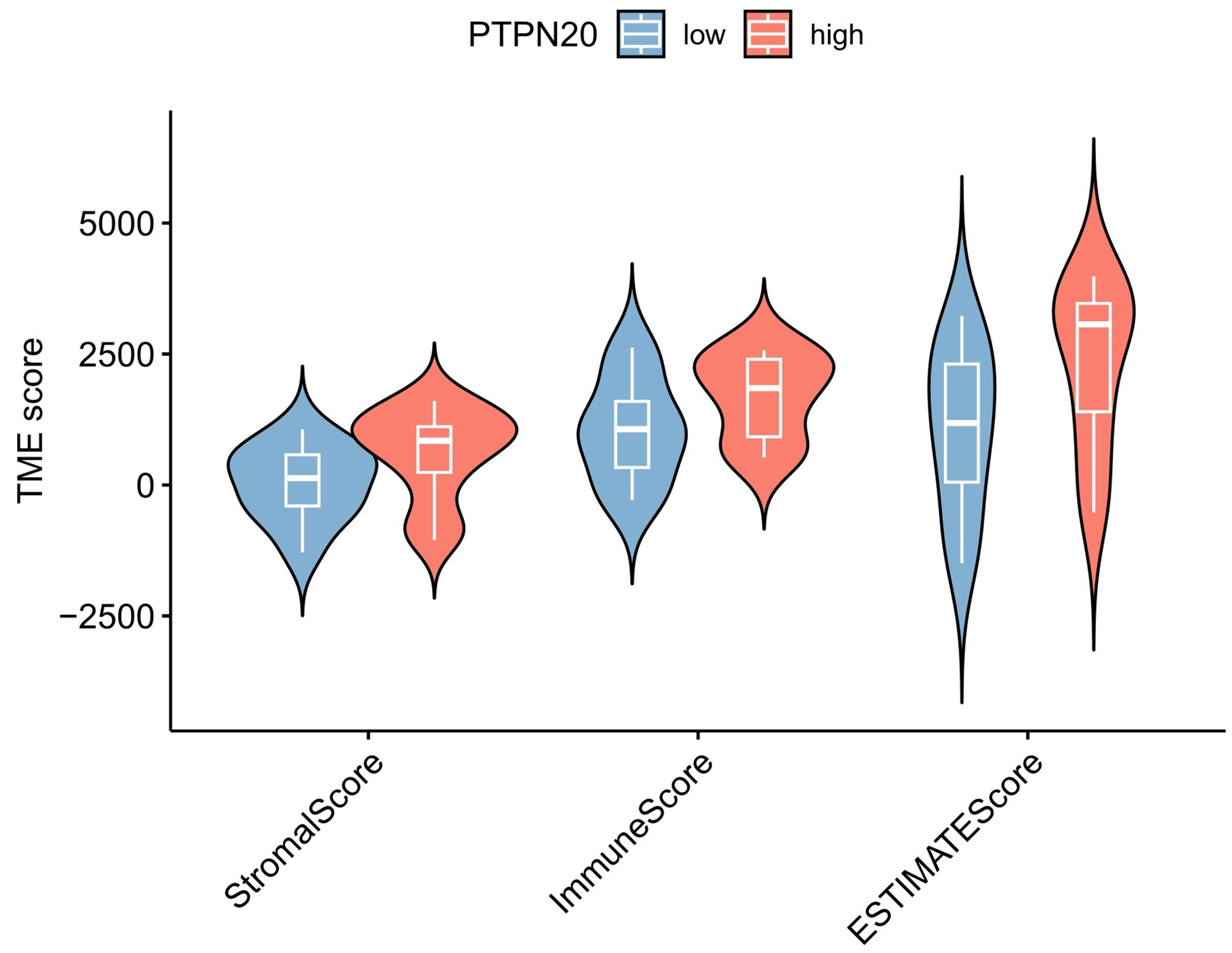

Supplement: Supplementary Figure 2 — The relationship between PTPN20 expression and tumor microenvironment scores. The PTPN20 low expression group had lower immuneScore, StromalScore, and ESTIMATEScore compared to the high expression group. [file Image_2.tif]
